# Trust-Based Relational Intervention^®^ (TBRI^®^) Impact for Traumatized Children—Meaningful Change on Attachment Security and Mental Health after One Year

**DOI:** 10.3390/children11040411

**Published:** 2024-03-29

**Authors:** Monika Misevičė, Lina Gervinskaitė-Paulaitienė, Sigita Lesinskienė, Izabelė Grauslienė

**Affiliations:** 1 Clinic of Psychiatry, Institute of Clinical Medicine, Faculty of Medicine, Vilnius University, 01513 Vilnius, Lithuania; sigita.lesinskiene@mf.vu.lt; 2 Institute of Psychology, Faculty of Philosophy, Vilnius University, 01513 Vilnius, Lithuania; lina.gervinskaite@gmail.com (L.G.-P.); izabele.grausliene@fsf.vu.lt (I.G.)

**Keywords:** attachment, middle childhood, traumatized children, Trust-Based Relational Intervention

## Abstract

Children from vulnerable backgrounds often have insecure attachment or disorganized attachment, which are related to psychological troubles, and such children need interventions to help them heal. The attachment system reorganizes in middle childhood, and other important adults play a considerable role in children’s lives. Thus, it is essential to weigh the impact of psychosocial interventions, while the main focus of the intervention is the staff member’s direct work with the child through a trusting relationship. The primary purpose of this study is to investigate whether children’s attachment security and mental health outcomes change after participating in a trauma-informed, attachment-based, Trust-Based Relational Intervention (TBRI) provided in a daycare center. It was a case-series study involving twelve children aged 8–11 years. The child attachment interview (CAI), CBCL/6-18, TRF/6-18, and clinical interviews for parents and children were used, measuring the change between the TBRI implementation in the daycare center and after one year. For ten participants, we noticed an improvement in mental health; for seven participants, security scales improved; for two participants, their disorganized attachment changed into insecure–dismissing. We have preliminary evidence that vulnerable children may benefit in terms of attachment security and mental health from the trusting relationship that staff build using the TBRI.

## 1. Introduction

Already more than fifty years after the pioneer of attachment theory, John Bowlby, postulated that experience with attachment figures in childhood could influence a person’s future life, scholars are continuing the research, trying to measure the impact of attachment and capture the correlates between attachment and possible psychological or psychiatric disturbances in childhood, adolescence, and adulthood. Attachment relationships influence the formation of possible psychopathology in connection with other risk factors [1,2,3,4]. Despite different research designs and measures of attachment, we have a clear understanding of the links between attachment and cognitive, social, and emotional development; school adjustment; self-awareness; emotional regulation; and peer relationships [2,5,6,7,8,9,10,11,12].

Our study focused on middle childhood when, according to Kerns et al. [13,14], there is an emergence of metacognition, a child’s personality is maturing, and social life is changing; thus, alterations in a child’s attachment system also occur. The intensity with the primary attachment figures is weakening, and the character of these relationships is changing [15,16]. Also, at this developmental stage, some other adults, such as grandparents or teachers, can be used in some contexts as attachment figures [17]. However, little is known about how these meaningful relationships with other adults, such as teachers, can influence a child’s attachment system [18]. Until now, we have only found studies where scholars are trying to measure the impact of parenting interventions on child development or attachment, and very often, those interventions happen in early childhood [19,20]. However, we found no long-term (more than six months) studies trying to measure the impact of interventions on a child’s attachment security where the main intervention agents would not be the primary caregivers but rather other important adult people or therapists. We do know from the research with adults that therapeutic relationships and emotional support from other persons can enhance attachment security [21,22]. Therefore, we set a goal and expected that a therapeutic relationship with daycare center staff members could also influence children’s attachment security.

We do know from current research that children with traumatic childhood experiences in their past usually develop various disturbances in different areas of functioning [23,24,25,26,27,28]—emotional and behavioral regulation, self-identity and awareness, and attention regulation. Thus, such children need a targeted and complex therapeutic intervention that tries to respond to their specific needs and has to be focused on increasing safety, self-regulation, self-reflective information processing, integration of traumatic experience, engagement into attachment, and strengthening of positive emotions [29]. Trust-Based Relational Intervention (TBRI) was also created to respond to the needs of children with hardships in their past, so the researchers have chosen to implement this intervention in a daycare center and to measure its impact. Summarizing all the research that has already been conducted to measure the impact of TBRI, there are several published studies in which TBRI or its elements were tested under specific conditions and were found to improve outcomes for traumatized children [30,31,32,33,34], but there is only one study where the impact was measured on the child’s attachment system. In one study, it was found that children who attended a daycare camp for three weeks that was based on TBRI exhibited improvement in attachment-related behaviors. However, until now, no study has measured the impact of TBRI on a child’s attachment system using an observation method instead of questionnaires, trying to capture the change in the mental representations of attachment. As the main principle of TBRI is creating and strengthening trusting relationships and a secure attachment, it was thought to be valuable to evaluate this possible change in a child’s attachment system after receiving TBRI. Thus, this study is the first to assess possible changes in the attachment system following TBRI using a highly valid measure of attachment in middle childhood—the child attachment interview (CAI).

Summarizing all the background, the primary purpose of this study is to investigate whether traumatized children’s attachment security and mental health outcomes change after participating in a trauma-informed, attachment-based, Trust-Based Relational Intervention (TBRI) provided in a community daycare center for one year. Our main hypotheses are as indicated below:

1. Children attending the daycare center have adverse childhood experiences, and they will have symptoms of different emotional and behavioral disturbances;
2. Positive changes in children’s mental health after 1 year will be observed;
3. In CAI, subscales indicating the security of attachment will shift towards more secure.

## 2. Materials and Methods

### 2.1. The Setting of the Study

We chose to measure the impact of TBRI in a non-governmental social daycare center (there are more than 400 such centers in the country) located in the center of Vilnius. It was a targeted sample, and we chose this particular daycare center because the staff were willing to cooperate with the researchers. The staff had indicated that their children were clients with emotional and behavioral challenges, and they were, therefore, looking for a specialist who could teach them how to respond to their clients’ needs. When the leader of the center was introduced to the TBRI method, she was ready to implement it in her center.

Usually, children are referred to the center by social workers, or the families themselves ask for their children to be taken in (this often happens when families already have older children attending the center). Most often, the center accepts children from socially vulnerable families—those who are already receiving help from social workers because of low income, unemployment, illness or dependency in the family, or poor parenting skills. As a rule, attendance at a daycare center is recommended for families who need help with their parenting tasks; the children come after school, do their homework at the center, and take part in various activities that teach them socially acceptable communication skills, self-regulation, and emotional awareness. There are also leisure activities aimed at promoting healthy lifestyles (e.g., sports, camping) and learning to choose meaningful leisure activities (e.g., going to the theater, excursions). The ratio of staff to children is quite low, approximately 0.2–0.5, but the staff are supported by adult volunteers.

All the parents whose children, aged 8–11 years, had attended the daycare center during the study year were informed about the study and invited to participate. Twelve participants (female = 3, male = 9) whose parents gave informed consent participated in the study. Accordingly, 12 participants’ mothers participated.

### 2.2. TBRI and Its Implementation in the Daycare Center

TBRI is a holistic, attachment-based, trauma-informed, evidence-based intervention grounded on neurodevelopmental knowledge and designed to meet vulnerable children’s complex needs [31,35]. TBRI is not a new psychotherapeutic approach but rather a caregiving model, which must be used in daily life and becomes a communication and lifestyle model for the adults that practice it. The training for TBRI is composed of teaching the theory about attachment, the brain, the impact of trauma, child development, sensory processing, reflecting on an adult’s feelings, relationship history, as well as a practical learning of communicative tips with a child. The intervention is formed of 3 main principles: Connecting, Empowering, and Correcting. A summary of the elements of each principle is given in Table 1 [35]. These principles consist of various practical elements whose goal is to seek that a child could feel more safe and secure.

Six staff members of the daycare center received 22 h of training on TBRI from the principal researcher M.M, a certified practitioner in TBRI. Afterwards, they had monthly case-analysis sessions according to TBRI for one year. In this daycare center, the employed staff members are supported by adult volunteers; therefore, the volunteers received 8 h of introductory training in TBRI and, afterwards, had a 2 h meeting to remind them of the essential principles of TBRI.

### 2.3. Assessment

Assessments were scheduled at baseline (T1), after six months (T2), and after 1 year (T3) of attending the daycare center. In Table 2, the structure of the assessment procedure is given.

#### Questionnaires

1. ASEBA (Achenbach System of Empirically Based Assessment) questionnaire Child Behavior Checklist for Parents (CBCL/6-18) and Teachers (TRF/6-18) standardized Lithuanian translations were used [36]. CBCL/6-18 and TRF/6-18 questionnaire statements are separated into eight possible syndromes: anxiety/depression; withdrawness/depression; somatic complaints; social problems; thought problems; attention problems; rule-breaking behavior; and aggressive behavior. After transferring the scores into the profile, it can be determined which score is in the normal, borderline (93–97 percentiles), or clinical range (98 percentile and above), and the range scores differ for boys and girls. In this paper, we will present the results from the problem scales of the CBCL/6-18 and TRF/6-18 questionnaires.

2. A semi-structured clinical psychiatric interview for parents (only mothers participated) was used. The main goal of the interview was to obtain information about possible risk factors for mental disorders, possible adverse childhood experiences, the child’s development history, actual mental health functioning, and actual possible symptoms of mental disorders. This interview was developed by the principal researcher, a child psychiatrist, according to the guidelines indicated in JM Rey‘s IACAPAP e-Textbook of Child and Adolescent Mental Health [37]. The interview consisted of 17 open questions with clarifying subquestions if needed. M.M. conducted all the interviews with the parents, and the answers were written down on paper and analyzed afterwards.

3. Child Attachment Interview (CAI) for children was used at T1 and T3. The CAI was developed by Shmueli-Goetz and her colleagues from the Anna Freud National Centre for Children and Families in London as a measure designed to assess attachment in middle childhood and adolescence [38]. It is a direct interview where a child is asked to describe his/her relationships with the persons he/she names in the first question about his/her family members. The CAI is video recorded because it is also crucial to justify the coding on non-verbal behavior. The CAI consists of 19 questions about the family, self-description, a description of the relationship with each attachment figure, the episodes when they were crossed with the child, and the situations where the child felt upset, was ill, was hurt, experienced separations, or experienced loss. The CAI requires that the child be interviewed by a stranger to awaken the attachment system. After the transcription of the CAI, it can be coded by a certified coder where a score is given to the following nine scales: emotional openness, balance of positive and negative references to attachment figures, use of examples, preoccupied anger, resolution of conflicts, idealization, dismissal, atypical/disorganized behavior, and overall coherence. Emotional openness, balance of positive and negative references (to attachment figures), use of examples, resolution of conflicts, and overall coherence scale scores of 5 and above to 9 indicate attachment security, and scores below 5 indicate insecurity. Finally, the attachment organization is determined; a child can be classified as secure, insecure–dismissing, insecure–preoccupied, and disorganized. A different attachment classification is designed for all attachment figures, and for disorganized children, a second alternative classification of secure, dismissing, or preoccupied is assigned. In this study, the Lithuanian translation of the CAI was used [39]. The CAI is valued as one of the measurements that has the best psychometric characteristics to measure attachment in middle childhood [40,41,42].

In our study, in order to keep the requirement of the CAIs, the children were interviewed by the principal researcher herself at T1 (when the children did not know her) and by the research collaborators who had the 3 h training on how to conduct the CAI. The interviews took place in the daycare center’s consulting room in private and were video recorded. Afterwards, the interviews were transcribed by M.M. and collaborators and coded by an independent certified CAI coder. The second certified CAI coder coded thirty percent of the interviews to test the reliability of the coding, and the two coders were blind to each other’s scores. There was 100 percent agreement between the two coders on the attachment classification. The intraclass correlation coefficients (ICC) between the two coders were substantial for eleven subscales, ranging from the lowest ICC for one subscale of 0.64 (resolution of conflicts) to the other subscales of 0.70 to 0.92. The median ICC indicates robust agreement between the two coders; for all scales, it was 0.86.

4. Clinical mental status examination interviews with a child occurred at T1, T2, and T3. M.M. structured this interview according to the guidelines indicated in JM Rey’s IACAPAP e-Textbook of Child and Adolescent Mental Health [37]. It consists of 22 questions with subquestions if needed. After the interview, the researcher filled out the form about the child’s mental status—his capacity to concentrate, communicate, actual mood status, etc.

5. Questionnaires and semi-structured interviews about the use of TBRI were used at T2 and T3. The principal researcher developed this questionnaire. It consists of 4 sections: the first three encompass statements about three main structural parts of TBRI: Connecting principle (12 items), Empowering principle (7 items), and Correcting principle (13 items). The staff members had to choose how often they used each intervention element: never/sometimes/often/always. The 4th section of the questionnaire has 3 open questions, but in this article, we will present only the results from the quantitative part of this questionnaire.

### 2.4. Data Collection Time and Data Processing

Data collection started in August 2019 and finished in August 2021; this period included the COVID-19 pandemic and quarantine period in the country. The first nine participants who joined the study in August 2019 had approximately two months of not attending the daycare center. The staff maintained a minimal relationship with them at that time. The last three participants who joined the study in August 2020 received more individual time from the staff than group-work time because of the quarantine requirements. These three participants received services for 1 year, while the others received services for 10 months due to quarantine.

M.M. analyzed all the data from the CBCL/6-18 and TRF/6-18 questionnaires and the interviews with the mothers and children. After careful analysis, if it was appropriate, a mental disorder diagnosis was assigned according to the International Statistical Classification of Diseases and Related Health Problems, 10th Revision [43]. The data from T2 were not included in the final analysis because of the lack of questionnaires from the teachers (8 were lacking out of 12). As at T3, 50% of the questionnaires were lacking from the teachers; they were not included in the final analysis, so the change between T1 and T3 was measured using only the questionnaires completed by the mothers.

#### Statistical Analysis

All analyses were performed using R (version 3.6.2) and R Studio (version 1.3.959) software. Continuous variables are expressed as the mean ± SD. These were checked for normal distribution using the Shapiro–Wilks test. Since sample size was small and normality assumption was rejected, paired comparisons of continuous variables were performed using the Wilcoxon signed-rank test. A *p* value < 0.05 was considered statistically significant. Power was checked using G Power 3.1.9.6software. Statistical analyses were performed on the data obtained from the CBCL/6-18 and TRF/6-18 questionnaires. The effect size was calculated by first taking the difference between the T1 and T3 scores for each subscale of the CBCL/6-18 and anologically for the use of the TBRI elements at T2 and T3 and then dividing the mean of this difference by its standard deviation. Intraclass correlation coefficients (ICC) were calculated to test the reliability of the coding of the CAI.

## 3. Results

### 3.1. Descriptive Data

All children had two or more adverse childhood experiences in their past: physical abuse, physical and emotional neglect, household violence, parents with alcohol dependencies, temporary removal from biological parents. Nine out of 12 participants, after careful analysis of the data from the interviews and the questionnaires, were diagnosed with one or two diagnoses (Table 3). The child psychiatrist saw none of these children before the study. One child had a consultation with a neurologist because of tics.

The prevailing symptoms of emotional disturbance were anger outbursts (for several children with physical aggression) at home, at school, or in the daycare center; suicidal thoughts and threats; various phobias; depressive behavior and mood; and withdrawal (most often noticed by the teachers, not by the mothers). For the girl diagnosed with elective mutism, the symptoms were revealed by her teacher, and at T3, the symptoms of mutism were strongly expressed in the CAI—she suddenly stopped talking and then only agreed to write down the answers; she did not speak until the end of the interview.

#### Attachment Organization and Its Links with Emotional or Behavioral Symptoms

Out of the twelve participants, no secure attachment was found (see Table 3). The classification of the attachment organization was the same for the mother and the father figure for all participants except one boy; he was classified as insecure–dismissing for the mother and insecure–preoccupied for the father. Of the children classified as disorganized at T1, one girl had no clinical symptoms; neither her mother nor her teacher reported any possible problems or symptoms. This girl attended the daycare center the least—she had been present only nine days per year. Her scoring of the CAI did not change at T3; she was also classified as disorganized at T3. The two other participants who were disorganized at T1 had been diagnosed with other childhood emotional disorders (F93.8) and disturbance of activity and attention (F90.0). One boy with disorganized attachment had nonorganic enuresis. Other possible emotional or behavioral disturbance’s symptoms were not reflected on the questionnaires and the interview with the mother, though this boy’s teacher noted attention problems. When comparing the CBCL scales of the children with disorganized and insecure–dismissing attachment at T1, the results showed that the children with disorganized attachment had higher scores on most scales. However, the differences were not statistically significant.

### 3.2. The Change after 1 Year

Five participants out of twelve demonstrated a marked improvement in behavior and emotional functioning, and for them, an improvement in the security indicating scales in the CAI was also noticed. One participant‘s attachment classification changed from disorganized to insecure–dismissing. For four others, we saw an improvement in the security scales of the CAI. The improvement was less expressed for the other five participants, but for three of them, some scales indicating security of attachment in the CAI were improved. One boy‘s attachment shifted from disorganized into insecure–dismissing, but clinically, the improvement was minimal. Summarizing the results of the CAI at T3 for seven participants, a slight improvement in the scales indicating security was noticed (see Table 4).

At T3, lower scores were noticed on most scales of the CBCL/6-18 questionnaire compared to T1, but only three subscales revealed statistically significant differences (see Table 5).

#### Participants with the Attachment Organization Change

The case of John (pseudonym). This boy’s improvement at T3 was very noticeable. At T1, this child was eight years old. He had anger outbursts with aggression, problems at school (difficulties sitting still during the lessons; behavioral problems and conflicts with a teacher; writing difficulties), attention deficit, suicidal thoughts, and threats. He was diagnosed with other childhood emotional disorders (F93.8) and disturbance of activity and attention (F90.0). This child had been examined previously in the pedagogical–psychological service for problems at school. There, it was confirmed that he had attention problems and emotional problems; consequently, he was assigned to have a teacher’s assistant during some lessons. The mother revealed that, during her pregnancy, she used alcohol in heavy portions and smoked; after birth, she reported physical and emotional neglect and household violence. When John was five years old, his mother along with him and his sister were brought out from their house to the crisis center for approximately six months to be secure from the father’s violence while drunk. Both parents are addicted to alcohol, but for several years, they have adhered to sobriety. At T3, John’s disorganized attachment classification changed to an insecure–dismissing and the balance, overall coherence, and examples scales improved. According to his mother, this boy’s attachment organization change is consistent with his clinical improvement; at T1, the CBCL/6-18 subscales of anxiety/depression and rule-breaking behavior were at clinical range and, at T3, were at normal or borderline range. According to the teacher, at TRF/6-18, the withdrawn/depressed, thought problems, and attention problems scales remained at the clinical range. It is important to note that, in spring 2020, the boy was learning at home online because of the COVID-19 quarantine requirements, so the teacher filled out the questionnaire at T3 after the boy returned to the school desk for approximately 1.5 months only. When this shift towards better mental health was noticed for this boy, it was perceived that he had several supportive factors during the study year, which could contribute to this improvement; he attended weekly individual psychological sessions (the staff noticed his poor mental status and offered psychological counselling in addition to the usual daycare center attending). Also, he had weekly, stable individual time with an adult volunteer with whom he had an excellent relationship. Likewise, during the COVID-19 quarantine, the family moved from the capital city to the village, and it seemed to be a good experience for them. Also, it was noticed that this boy attended the center most often compared to others—93 days per year—while the mean of attendance was approximately 63 days per year.

The case of Tim (pseudonym). Tim, at T1, was eight years old. His mother complained that he often had nocturnal enuresis episodes and a fear of the dogs (though it was not enough of a disturbing symptom to confirm the possible phobia disorder). At CBCL/6-18, all the subscales were in the normal range. At TRF/6-18, likewise, all the subscales were in the normal range, though the teacher added by handwriting that he had problems with attention and concentration. This boy also had several adverse childhood experiences in his past—he had been abused physically by his mother and witnessed physical violence by his stepfather against his mother. At five years old, he was taken from the home and placed in foster care for nine months because of his mother’s lack of maternal competencies. This boy was classified as disorganized at T1, and he also changed into insecure–dismissing at T3. However, his shift in the CAI did not correlate with the mother’s report. On the contrary, the mother had reported more problems at T3—at CBCL/6-18, withdrawn/depressed and thought problems were at the clinical range. Disappointingly, the teacher’s report at T3 was not received.

### 3.3. The Use of the TBRI Elements

Table 6 gives the general trends as measured by the questionnaire about using the TBRI elements.

The questionnaires were filled out by four staff members who worked directly with the children. We can notice that, already at T2, the staff used the majority of the Connecting elements often or always, and at T3, more Connecting elements were used always than at T2. At T3, more Empowering elements were used often than sometimes. We can see that more Correcting elements were used always at T3 than at T2.

## 4. Discussion

In our study, we aimed to investigate whether children’s attachment security and mental health outcomes change after participating in a trauma-informed, attachment-based, Trust-Based Relational Intervention (TBRI) provided in a daycare center for one year. The results showed improvements in several aspects of mental health and attachment security for ten children after participation in TBRI. However, we acknowledge that our findings are based on a small sample, and therefore, we are not in a position to make broad generalizations about this group of children. Therefore, some of the following discussion reflects our thinking based on years of experience of working with such children.

Among attachment researchers, a strong tendency exists to argue that the attachment system is more malleable than stable and could be influenced by different relationships [44,45,46]. Therefore, this understanding—that relationships matter for attachment—was one of the main theoretical pillars of this study. This was thought to be even more important in middle childhood, when adults other than primary caregivers become more significant. This focus seemed even more important to us because children often stay in these daycare centers for an extended period (sometimes even until adulthood), so the staff members form close relationships with them. In addition, these children come from socially disadvantaged families, often with dependency, unemployment, financial or housing problems, and single-parent families, so the parents are often overwhelmed by a variety of concerns and tend to be difficult to reach for parenting interventions, as some studies have also shown [47,48]. So, in this study, we looked at the possible changes that other children’s relationships with a trusted adult might have on their attachment system.

Taking into account the limitations of the study design, we observed positive changes in the mental health of most participants after TBRI. According to the data obtained from the CBCL/6-18 questionnaires, there were lower scores at T3 on most scales, and on three scales, we saw statistical significance: anxiety and depression; social problems; and aggression. We expected that, in the CAI, the subscales indicating security would improve, and for seven out of the twelve participants at three or more security subscales, the improvement was noticed. One subscale—overall coherence—did improve for all of those seven participants. For two boys, their attachment classification changed from disorganized to insecure–dismissing. Although this is only a preliminary result due to the limitations of this study, it is a relevant finding because the appearance of disorganization in the CAI is understandable as a qualitatively different trait that distinguishes disorganized attachment from other types. This shift from the disorganized attachment category to the insecure–dismissing category was observed in the absence of parental intervention, which is important for future research. As disorganization is very much linked with various disturbances in childhood [49,50,51,52,53] and can even be transmitted intergenerationally [54], likewise, it is understood that disorganization is least likely to transition to security in the absence of intervention [44]. Here are data about early (until ~ 6 years old) interventions positive effect on children’s disorganization focused on f primary caregivers (biological or adoptive parents) [55,56,57]. We also have data on attachment-based interventions that focus on improving attachment security in the population of foster or adopted children [57,58,59,60], although all of these interventions focus on improving the parenting skills of foster or adoptive parents. There is one study that reported on attachment-informed intervention’s positive impact on the behavior of deprived primary school children [61]. However, to our knowledge, there has been no published research on the effect of interventions on disorganization in children in middle childhood. But there is evidence that psychosocial interventions for institutionalized children aged 6 months to 4 years have a positive effect on attachment security, and the main pillar of the intervention is the creation of a responsive relationship between staff and children [62,63].

Our findings are in accordance with the theoretical background that other significant adults can play a crucial role in children’s attachment. Although preliminary, our findings are consistent with the conclusions of a report by the National Scientific Council on the Developing Child at Harvard University [64] (p.5)—they have stated that what helps a child be resilient in the face of significant adversity is “the availability of at least one stable, caring, and supportive relationship between a child and the important adults”. It is mentioned that this relationship begins with the primary caregivers but includes other adults as well [64].

As measuring attachment using the CAI requires many resources (an interview by a stranger, then transcription, and a complex coding system), there are studies that measure attachment using the CAI [39,65,66], though to our knowledge, no studies measured attachment organization change using the CAI with a pre- and post-therapeutic intervention design. A meta-analysis [44] investigated attachment stability and change in early childhood (until ~ 6 years), and the authors stated that insecure and disorganized attachment styles are prone to change. We know from research that the attachment pattern changes at middle childhood [15,16,17,67], and there is a longitudinal study reporting on attachment change from 10 years of age to early adolescents using the Attachment Interview for Childhood and Adolescence (AICA)—they found rather high stability for secure and dismissing attachment, whereas the preoccupied category and the unresolved state of mind (analogous to disorganized in the CAI) were less stable [68]. Although our findings should be treated with caution, they add to the understanding that changes in attachment are possible in this age group.

The descriptive data from this study showed that the children who participated had adverse childhood experiences in the past and not only did they appear to have some symptoms of emotional and behavioral disorders but the majority of them already fit into psychiatric diagnostic categories. Also, they were found to have either an insecure or disorganized attachment style, and there were no secure children in this group. Therefore, as was hypothesized, such children need targeted intervention; this study’s findings suggest that TBRI could be very helpful, and TBRI was easily accepted and used by the staff.

This was the first study in the field of TBRI to look at changes in a child’s attachment system following an intervention using an observable and highly valid measure of attachment, the CAI. As the core principle of all TBRI is built on the understanding and efforts that a child could feel more secure with a safe adult and heal with the help of a trusting relationship, it is logical to expect that this intervention can help improve the security of attachment for the child. It is highly recommended that TBRI be used by the parents themselves at home with the children because then we would expect even more significant change for the children. Probably, that is why the majority of the research on the impact of TBRI is when intervention is applied by adoptive parents at home [33,34,69]. Our results are in line with most previous research on TBRI, which was made to explore the therapeutic camp effect for traumatized children [30,32]. The conclusions of our study should be taken with caution, but they are promising. They suggest that TBRI delivered by staff is likely to make a tangible difference to children.

As our study has limitations and we can only observe a tendency that the TBRI provided by the staff can be helpful for children’s attachment and mental health, we would observe that, for future perspectives, a study with a more rigorous design would be needed. It would be valuable to follow such children over several years using the same instrument to measure attachment and to capture any changes in the attachment system and possible reasons for them. We have observed in our practice that daycare center staff form stable relationships with the children and their families and can thus become a resource for these families. It would, therefore, be interesting to explore the staff–child relationship and its relation to TBRI practices in the design of the qualitative study.

## 5. Limitations

Case-series study design has limitations: we had no control group, so we can only observe the tendency and preliminary evidence that a change occurred after the TBRI^®^, but future research with a more rigorous study design would be needed to confirm this trend.

Another limitation occurred because of the COVID-19 pandemic. Because of the quarantine requirements in the country, the participants differed slightly in how they received services in the daycare center. The first nine participants had approximately two months of interruption in attendance, and the last three children who joined the study in 2020 had more individual time with the staff than group work.

One more limitation is that most of the questionnaires were missing from the teachers at T2 and T3. This can be explained by several points. In most cases, the mothers were asked to give the questionnaires to the teachers themselves. As it has been mentioned that the children came from socially deprived backgrounds, the failure to hand over the questionnaires can be explained by the fact that these families face various difficulties, and the questionnaires were the last priority for them. It may also be that the teachers did not have the confidence to answer the questionnaires, especially at T2 and T3, as there was no live contact between the teachers and the researcher.

## 6. Conclusions

This paper aimed to investigate whether children’s attachment security and mental health outcomes change after participating in a trauma-informed, attachment-based, Trust-Based Relational Intervention (TBRI) provided in a daycare center. This study’s novelty was that we investigated and searched for the possible impact of the staff member’s relationship with the child on their attachment security, and there were no specific interventions with the parents. Considering the limitations of this study, the findings suggest that positive changes in children’s mental health and attachment security were observed after TBRI. For two participants, their disorganized attachment shifted to an organized classification after one year. As disorganization is associated with various disorders, not only in childhood but also in adult life, and although more research is needed to generalize beyond the reference group, this finding is promising. It can give meaning to the hard work of daycare center staff; their dedication is likely to benefit children with adverse childhood experiences, mental health, and attachment.

## Figures and Tables

**Table 1:** TBRI principles.

Connecting Principle	Empowering Principle	Correcting Principle
Engagement Strategies—teaching to connect with children, such as with eye contact, behavioral matching, and playful engagement. Mindfulness Strategies—training caregivers on awareness of what they bring to the interactions with children; also training on the importance of self-care.	Physiological Strategies—focus on the internal physical needs of a child (hydration, nutrition, and sensory needs). Ecological Strategies—focus on a child’s external environment (transitions, scaffolding, daily rituals) and learn self-regulation skills.	Proactive Strategies—designed to teach social skills to children during calm times. Responsive Strategies—provide caregivers with tools for responding to challenging behavior from children.

**Table 2:** Structure of the assessment procedure.

T1 (August–September 2019 or 2020)	T2 (February–March 2020 or 2021)	T3 (August–September 2020 or 2021)
Clinical interview with the child CAI with the child Clinical interview with a parent + CBCL/6-18 TRF/6-18 for teachers	Clinical interview with the child --- Clinical interview with a parent + CBCL/6-18 TRF/6-18 for teachers Questionnaire for the staff about the use of TBRI	Clinical interview with the child CAI with the child Clinical interview with a parent + CBCL/6-18 TRF/6-18 for teachers Questionnaire for the staff about the use of TBRI

**Table 3:** Descriptive data.

Age, Mean (SD)	Distribution by Gender	Adverse Childhood Experiences, Mean	Diagnoses According to ICD-10 AM			Daycare Center Attendance in Days, Mean, SD	Attachment Organization at T1
9.4 (0.99)	9 Boys 3 Girls	2	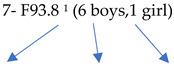			62.9; 32.2 ^2^	7 Insecure–Dismissing 1 Insecure–Preoccupied 4 Disorganized
			1 boy F93.8 + F90.0	1 boy F93.8 + F95.1	1 girl F93.8 + F98.0		
			1 boy—F98.0				
			1 girl—F94.0				

^1^ F93.8—Other childhood emotional disorders; F90.0—Disturbance of activity and attention; F95.1—Chronic motor tic disorder; F98.0—Nonorganic enuresis; F94.0—Elective mutism. ^2^ One participant only attended for nine days per year, which is why the SD value is so high.

**Table 4:** General trends of scoring change from T1 to T3 as coded at the CAI.

Participants ID	EO *_T1	EO_T3	Bal *_T1	Bal_T3	Ex *_T1	Ex_T3	Confl *_T1	Confl_T3	Coh *_T1	Coh_T3
012 **	4	2	2	4	2	3	2	3	2	3
013	4.5	5.5	5	5	4	5	5	5	4.5	5
014	2	2.5	2	3	1.5	2	3	4	2	2.5
016	1	2	3	3	1	2	2	3	1	3.5
08	5	6	4	5	4	4.5	4	5	3	4
09	4	5	3	4	4.5	4.5	4	4	3.5	4
018 **	1	2	2	3	1	1.5	3	3.5	1	2.5

* Eo stands for emotional openness; Bal—balance of positive and negative references to attachment figure; Ex—use of examples; Confl—resolution of conflicts; Coh—overall coherence. ** Attachment classification change from disorganized to insecure–dismissing was noticed for those participants.

**Table 5:** General trends of symptom changes as measured by the CBCL6/18 from T1 to T3.

Subscale	T1		T3		
	M	SD	M	SD	Effect Size
Anxiety/Depression	6.8	1.9	4.9 *	2.0	1.05
Withdrawn/Depressed	4.0	2.9	4.2	3.8	0.05
Social Problems	6.1	3.2	3.9 *	2.6	0.57
Thought Problems	3.8	2.9	2.2	1.9	0.61
Attention Problems	8.9	3.8	7.5	2.1	0.38
Rule-Breaking Behavior	4.8	2.4	4.2	2.2	0.26
Aggressive Behavior	11.9	4.4	7.2 *	3.3	1.22

* *p* value < 0.05.

**Table 6:** The use of the TBRI.

	T2		T3		
	M	SD	M	SD	Effect Size
Connecting Principle:					
Sometimes	1.6	1.3	1.25	0.5	0.58
Often	3.0	0.8	1.5	0.5	2.64
Always	1.4	0.5	2.7	0.9	2.92
Empowering Principle:					
Sometimes	2.6	0.9	1.3	0.6	1.44
Often	1.5	0.6	1.9	0.7	0.39
Always	2.0	0.8	2.0	1.0	0.5
Correcting Principle:					
Sometimes	1.7	0.7	1.0	0	1.29
Often	2.4	0.9	2.0	0.9	0.25
Always	1.3	0.5	2.3	0.7	1.41

## Data Availability

The data presented in this study are available on request from the corresponding author due to ethical restrictions and language barrier (Lithuanian).
